# Primary Tumor Resection Improves Survival for EGFR-TKI-Treated Patients With Occult M1a Lung Adenocarcinoma

**DOI:** 10.3389/fonc.2021.622723

**Published:** 2021-04-19

**Authors:** Fangqiu Fu, Zhexu Wen, Zhendong Gao, Yue Zhao, Yuan Li, Yang Zhang, Haiquan Chen

**Affiliations:** ^1^ Department of Thoracic Surgery and State Key Laboratory of Genetic Engineering, Fudan University Shanghai Cancer Center, Shanghai, China; ^2^ Institute of Thoracic Oncology, Fudan University, Shanghai, China; ^3^ Department of Oncology, Shanghai Medical College, Fudan University, Shanghai, China; ^4^ Department of Pathology, Fudan University Shanghai Cancer Center, Shanghai, China

**Keywords:** lung adenocarcinoma, M1a, primary tumor resection, EGFR-TKIs, survival

## Abstract

**Background:**

The role of primary tumor resection in occult M1a lung adenocarcinoma remains unclear, especially for patients receiving targeted therapy. The purpose of this study is to assess the effect of primary tumor resection on overall survival (OS) in lung adenocarcinoma patients with occult pleural disseminations receiving targeted therapy.

**Methods:**

Lung adenocarcinoma patients with intraoperatively-confirmed occult pleural dissemination (M1a), who hospitalized in the Department of Thoracic Surgery in Fudan Shanghai Cancer Center from May 2008 to December 2017 and received EGFR-TKIs therapy, were enrolled. Log-rank tests were used to compare the survival differences between groups.

**Results:**

34 patients receiving EGFR-TKIs were enrolled. The majority of them were never smokers (29/34, 85.3%). Among the enrolled patients, 20 (58.8%) patients underwent primary tumor resection, while 14 (41.2%) patients not. There was no distributional difference of baselines between patients undergoing and not undergoing primary tumor resection. Further analyses demonstrated that the patients undergoing primary tumor resection had a prolonged OS compared with those not (log-rank *P*= 0.042). The 2-year and 5-year OS for patients receiving primary tumor resection and EGFR-TKIs was 90.0% and 60.1%.

**Conclusions:**

Primary tumor resection was associated with improved survival in patients with occult intraoperatively-confirmed M1a adenocarcinoma receiving EGFR-TKIs.

## Introduction

Lung cancer is the deadliest malignancy worldwide, accounting for the largest number of new cancer cases and cancer-related deaths (1). Stage IV lung cancer is responsible for 45% of newly diagnosed lung cancer patients in Surveillance, Epidemiology, and End Results (SEER) Program population‐based registries (2). However, the 2-year and 5-year overall survival (OS) rate for stage IV non-small cell lung cancer (NSCLC) is only 17% and 6%, respectively (3). Over the past ten years, treatment for stage IV NSCLC has been revolutionized by the rapid development of targeted therapy. In the era of targeted therapy, lung cancer patients live a much longer life than before. Nowadays lung cancer seems to be a “chronic” disease thanks to the clinical application of targeted agents. However, patients will eventually develop drug resistance to targeted agents, and it is a critical issue to address methods improving survival of patients receiving target therapy. A previous study indicated that patients with initial disease progression in primary tumors accounted for 45% of progressed patients with targeted therapy (4). Moreover, in clinical practice, occult pleural dissemination is sometimes discovered intraoperatively. There is no consensus on whether we should perform primary tumor resection in that case. Therefore, we hypothesized that surgical resection of primary tumors could improve the survival of lung adenocarcinoma patients with occult pleural metastases in the era of targeted therapy.

Although surgery is not deemed as a treatment option, given the fact that therapeutic goals of stage IV disease have focused on optimization of quality of life and palliation, recent studies indicated local consolidative therapy were able to prolong progression-free survival and overall survival (OS) in stage IV NSCLC patients who received first-line systemic therapy (5, 6). Nevertheless, the role of primary lesion surgery in patients with occult M1a lung cancer receiving targeted therapy remains unclear.

To address this, we aimed to assess OS after primary tumor resection versus no resection in lung adenocarcinoma patients with occult pleural dissemination treated with EGFR-TKIs.

## Methods

### Patients

Selected patients with occult M1a lung adenocarcinoma hospitalized in the Department of Thoracic Surgery, Fudan Shanghai Cancer Center (FUSCC) from May 2008 to December 2017 were reviewed retrospectively. The inclusion criteria were (1) pathologically confirmed primary lung adenocarcinoma, (2) occult pleural dissemination and pathologically confirmed M1a intraoperatively by frozen section examinations, and (3) receiving targeted therapy toward *EGFR* mutation. Age, gender, smoking history, body mass index (BMI), receiving primary tumor resection or not, mutation status, EGFR-TKIs therapy, and survival data were collected. Primary tumor resection was defined as the surgical removal of primary lung cancer lesion, which was usually the largest or first appeared radiologically. The study was approved by the Institutional Review Board of Fudan University Shanghai Cancer Center (IRB#090977-1), and the protocol number of this study was IRB2008223-9.

### Mutational Analyses

The mutational analyses were conducted by the central laboratory of pathology in FUSCC using the resected primary tumor specimens. Genomic DNA was extracted for further amplification refractory mutation system.

### Statistical Analyses

Overall survival was calculated from the date of the diagnosis to the date of death or last follow-up, and death from any cause was considered as an event. Chi-square tests were used to evaluate the difference of categorized variables between patients receiving and not receiving primary tumor resection. The Kaplan-Meier method was used to analyze OS, and log-rank tests were used to compare differences between groups. Data were analyzed by SPSS (version 25.0; IBM Corp, Armonk, NY). All tests were two-tailed, and statistical significance was set at P < 0.05.

## Results

### Patient Characteristics

A total of 34 patients were enrolled in the study (**Table 1**). There are 22 (64.7%) females and 12 (35.3) males. A majority of patients (29/34, 85.3%) were never-smokers. Most of patients had cT1 and cT2 disease. 17 (50.0%) patients had cN0 disease, 5 (14.7%) had cN1, and 12 (35.3%) had cN2 disease. Among these patients, 20 (58.8%) patients underwent primary tumor resection, while the rest (14/34, 41.2%) not. Concerning firstly-used EGFR-TKIs, the most common agent was gefitinib (26/34, 67.2%), followed by erlotinib (6/34, 17.6%), icotinib (1/34, 2.9%), and osimertinib (1/34, 2.9%). Five (5/34, 14.7%) patients received osimertinib as the second therapy after developing drug resistance to gefitinib and erlotinib. Besides targeted therapy, 23 (67.6%) patients received platinum doublet chemotherapy, and 6 (17.6%) patients received radiotherapy.

**Table 1:** Clinicopathologic characteristics of patients with M1a lung adenocarcinoma.

Variables	Enrolled patients (N = 34)	
	N	%
**Age (years)**		
** ≤60**	21	61.8
** >60**	13	38.2
**Gender**		
** Male**	12	35.3
** Female**	22	64.7
**Smoking history**		
** Ever**	5	14.7
** Never**	29	85.3
**BMI**		
** ≤24**	23	67.6
** >24**	11	32.4
**cT**		
** cT1**	14	41.2
** cT2**	18	52.9
** cT3**	1	2.9
** cT4**	1	2.9
**cN**		
** cN0**	17	50.0
** cN1**	5	14.7
** cN2**	12	35.3
**Primary tumor resection**		
** Yes**	20	58.8
** No**	14	41.2
**Firstly-used EGFR-TKIs**		
** Gefitinib**	26	76.5
** Erlotinib**	6	17.6
** Icotinib**	1	2.9
** Osimertinib**	1	2.9
**Secondly-used osimertinib**		
** Yes**	5	14.7
** No**	29	85.3
**Platinum doublet chemotherapy**		
** Yes**	23	67.6
** No**	11	32.4
**Radiotherapy**		
** Yes**	6	17.6
** No**	28	82.4

BMI, Body Mass Index.

Comparisons were made between patients who received primary tumor resection and those who did not (**Table 2**). There were no distributional differences between two groups in age (*P*=0.477), gender (*P*=0.066), smoking history (*P*=1.000), body mass index (BMI, *P*=1.000), cT (*P*=0.207), cN (*P*=0.512), firstly-used EGFR-TKIs (*P*=1.000), secondly-used osimertinib (*P*=0.627), platinum doublet chemotherapy (*P*=1.000), and radiotherapy (*P*=1.000). Previous studies have revealed the prognostic value of clinical T and N descriptors in patients with operable lung cancer (7, 8), so we also investigated it in patients with intraoperatively-confirmed M1a disease. The results demonstrated that there was no difference in survival between patients with different cT (*P*=0.96) and cN (*P*=0.87) descriptors (**Supplementary Figure 1**).

**Table 2:** Clinicopathologic characteristics of patients receiving and not receiving primary lesion resection.

Variables	Patients receiving primary tumor resection (N = 20)		Patients not receiving resection (N = 14)		*P*
	N	%	N	%	
**Age (years)**					0.477
** ≤60**	11	55.0	10	71.4	
** >60**	9	45.0	4	28.6	
**Gender**					0.066
** Male**	10	50.0	12	85.7	
** Female**	10	50.0	2	14.3	
**Smoking history**					1.000
** Ever**	3	15.0	2	14.3	
** Never**	17	85.0	12	85.7	
**BMI**					1.000
** ≤24**	14	70.0	9	64.3	
** >24**	6	30.0	5	35.7	
**cT**					0.207
** cT1**	6	30.0	8	57.1	
** cT2**	13	65.0	5	35.7	
** cT3/4**	1	5.0	1	7.1	
**cN**					0.512
** cN0**	10	50.0	7	50.0	
** cN1**	4	20.0	1	7.1	
** cN2**	6	30.0	6	42.9	
**Firstly-used EGFR-TKIs**					1.000
** Gefitinib**	15	75.0	11	78.6	
** Erlotinib**	3	15.0	3	21.4	
** Icotinib**	1	5.0	0	0	
** Osimertinib**	1	5.0	0	0	
**Secondly-used osimertinib**					0.627
** Yes**	2	10.0	3	21.4	
** No**	18	90.0	11	78.6	
**Platinum doublet hemotherapy**					1.000
** Yes**	14	70.0	9	64.3	
** No**	6	30.0	5	35.7	
**Radiotherapy**					1.000
** Yes**	3	15.0	3	21.4	
** No**	17	85.0	11	78.6	

BMI, Body Mass Index.

**Figure 1:**
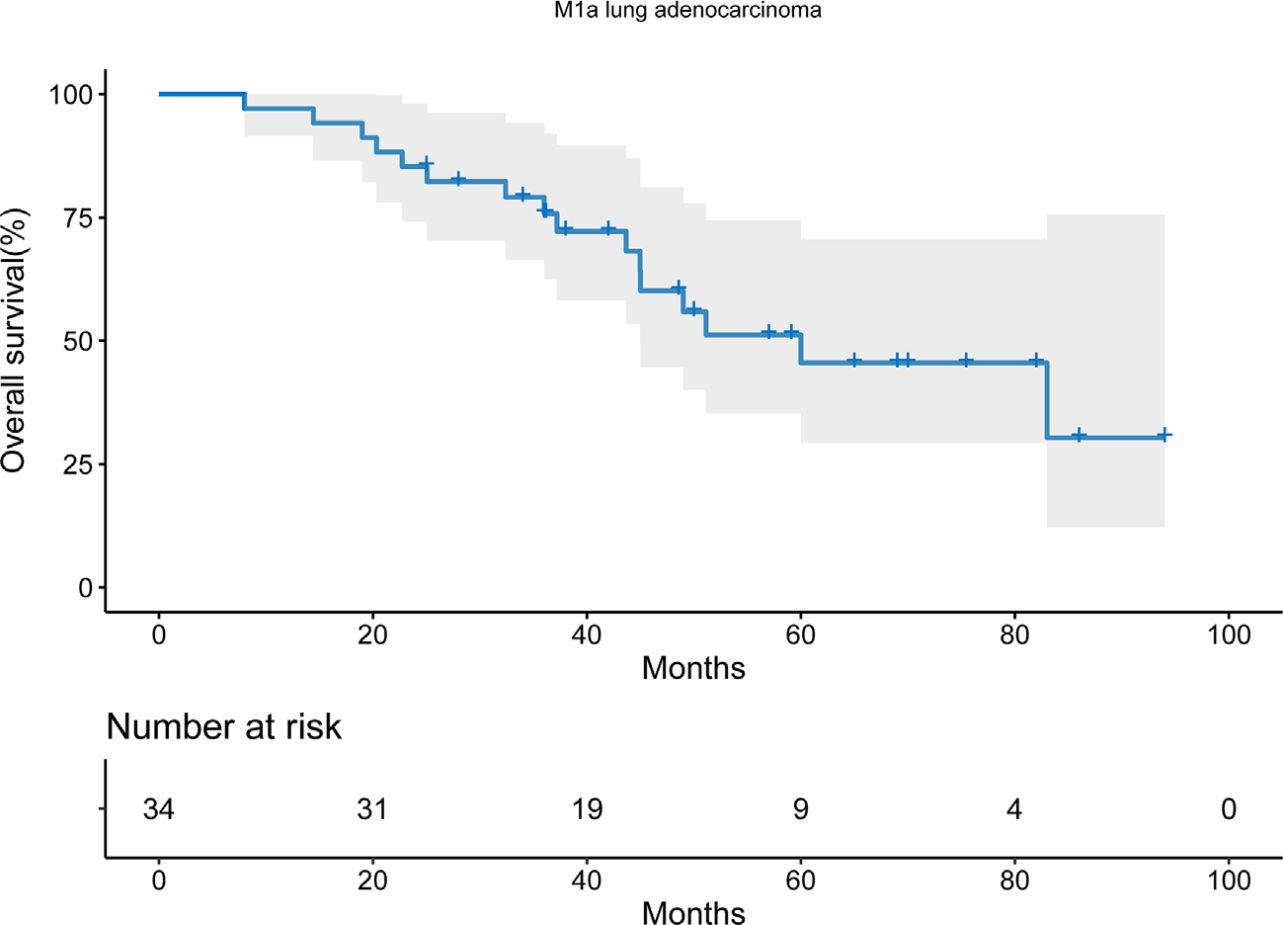
Overall survival of M1a lung adenocarcinoma patients receiving targeted therapy.

16 patients died during the follow-up period. With the median follow-up time of 65.0 months, the median OS was 60.0 months (95% confidential interval [CI], 33.4-88.6). The 2-year and 5-year OS was 85.3% and 45.5% respectively (**Figure 1**).

### The Association Between Primary Tumor Resection and Improved Survival

**Figure 2:**
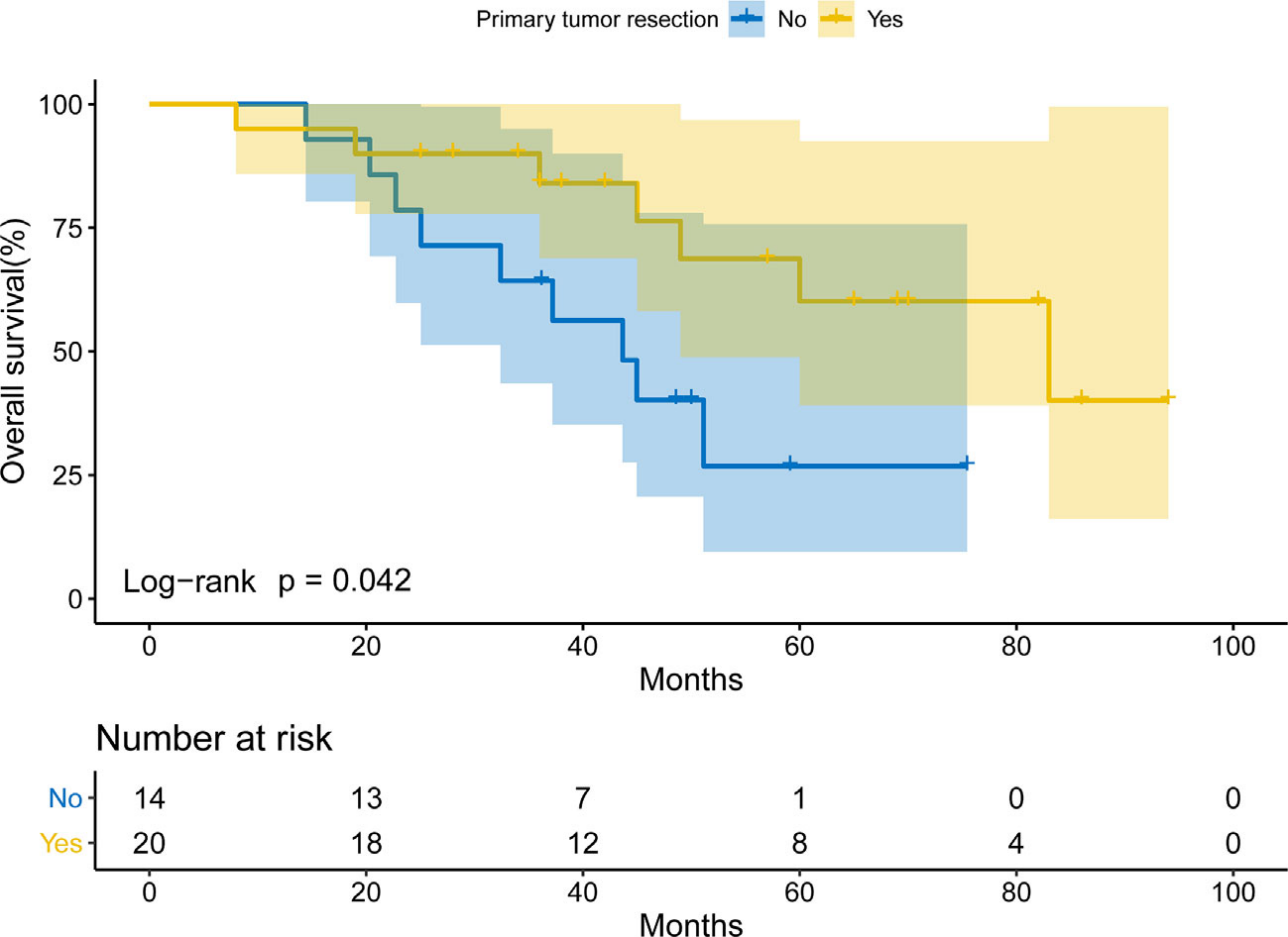
Overall survival of EGFR-TKIs-treated patients with intraoperatively-confirmed M1a lung adenocarcinoma receiving or not receiving primary tumor resection.

To investigate the prognostic role of primary tumor resection, Kaplan-Meier curves were used. The patients undergoing primary tumor resection had a prolonged OS compared with those not (log-rank *P*= 0.042; **Figure 2**). The median OS for patients receiving primary tumor resection and those not was 83.0 months (95% CI: 41.2-124.8) and 43.7 months (95%CI: 30.6-56.7). The 2-year and 5-year OS for patients receiving primary tumor resection was 90.0% and 60.1%, while the 2-year and 5-year OS for those not was 78.6% and 26.8%.

## Discussion

Currently, there was no consensus on whether to perform surgical resection to lung cancer patients with occult pleural disseminations. Chemotherapy or targeted therapy was recommended as initial treatments for stage IV NSCLC, and radiotherapy could also be considered if necessary, without the recommendation of surgical resection. Gomez and his colleges (5, 6) reported local consolidative therapy, including surgery and/or radiotherapy, could prolong progression-free survival and overall survival significantly in stage IV lung cancer after receiving first-line systemic therapy. However, only patients with stable disease and partial response to chemotherapy were enrolled, and most of the patients received platinum doublet chemotherapy as the first-line treatment. Besides, there were only six patients (6/25, 24%) receiving surgery of metastatic and/or primary sites in their study. Therefore, the actual effect of surgery in patients with stage IV lung cancer remains unclear, especially for patients receiving targeted therapy. In our study, we investigated lung adenocarcinoma patients with occult pleural disseminations receiving EGFR-TKIs and found out upfront primary tumor resection was associated with improved survival. To the best of our knowledge, it is the first study to reveal the association between primary surgical resection and improved survival in lung adenocarcinoma patients with occult pleural metastases in the era of targeted therapy. The study provided novel evidence for the treatments of occult M1a lung adenocarcinoma.

In clinical practice, pleural disseminations might be occult for some patients and was discovered intraoperatively. There is no consensus on whether we should perform primary tumor resection in that case. Since surgical resection can result in a 5-year survival rate of 30% to 50% in patients with metastatic NSCLC (9), and the role of thoracic surgery in the management of metastatic NSCLC attracts our attention. Theoretically, surgical resection of primary lesions could reduce tumor burden, which was considered to be associated with targeted drug resistance and prognosis of patients. In our study, primary tumor resection could significantly prolong the survival of patients receiving EGFR-TKIs (*P*= 0.042). The 5-year OS of patients undergoing surgical resection and those not was 60.1% and 26.8%. These results supported the conclusion that surgical resection could improve survival in lung adenocarcinoma patients with occult pleural disseminations receiving EGFR-TKIs.

With the improved disease response and control rate with targeted therapy, patients with advanced-stage lung cancer live longer. According to previous real-world studies (10), the median OS for *EGFR*-mutant metastatic lung adenocarcinoma patients treated with EGFR-TKIs was 30.9 months. Especially for Asian patients, our previous study more than half of patients with lung adenocarcinoma harbored *EGFR* kinase domain mutations (11). The targeted agent is an ideal treatment for them, but drug resistance will occur sooner or later. Our study might provide a potential way to slow down the drug resistance to EGFR-TKIs. Further randomized clinical trials are urged on the possible combined use of surgery and targeted therapy.

The survival benefit from surgical resection of primary tumors might be explained by the following potential mechanisms. Intratumor heterogeneity results in different subclones of tumor cells, some of which are resistant to targeted therapy or chemotherapy (12). After resection of the primary lesion, generally the largest of all lesions, the drug-resistant subclones could also be removed (13). Thus, in this scenario, patients may have a better drug response and longer survival. Another possible mechanism is that primary tumors seed circulating tumor cells *via* the bloodstream, resulting in micro-metastasis in distant sites (14). In that case, resection of primary tumors could slow the growth speed of micro-metastasis. Therefore, upfront surgical resection helped to maximize drug response of targeted therapy, suggesting a combination of surgical resection and targeted therapy might be an effective option.

In our study, surgical resection was performed before targeted therapy. Upfront surgery followed by targeted therapy could provide advantages in some ways. In our previous study, upfront surgery followed by adjuvant therapy may also provide favorable survival outcomes for selected patients with lung cancer (15). The resection of primary lesions spared patients from biopsies to confirm pathology and mutational status. Additionally, pathologic and mutational analyses based on surgically resected specimens were generally more precise than biopsy specimens. Therefore, upfront surgery followed by targeted therapy was feasible in clinical practice.

There are several limitations of this study. First, the number of patients seemed small. We only enrolled patients receiving EGFR-TKIs with intraoperatively-confirmed occult M1a lung adenocarcinoma, who have been hospitalized in the department of thoracic surgery. The multivariable analyses were inappropriate due to limited sample size. Nevertheless, the baselines of two groups were comparable, resulting in no confounding factors during the direct survival comparison by Kaplan-Meier method. Second, it is a retrospective study from a single institution, and selection bias was inevitable. Our results need to be validated in future multi-centered randomized controlled clinical trials. Third, progression-free survival was not calculated in the study, because some patients received surveillance in other institutions. Nevertheless, we believe that OS is a more important outcome and the study based on OS is more meaningful for patients. Fourth, the main agent of EGFR-TKIs in this study was gefitinib due to the use of histological data before the adoption of first-line osimertinib. However, the study provided a promising treatment for lung adenocarcinoma with occult pleural disseminations.

In summary, primary surgical resection improves survival of lung adenocarcinoma patients with intraoperatively-confirmed occult pleural metastases followed by EGFR-TKIs. Primary tumor resection might be a promising method for the treatment of patients with occult M1a lung adenocarcinoma receiving target therapy.

## Data Availability Statement

The original contributions presented in the study are included in the article/**Supplementary Material**. Further inquiries can be directed to the corresponding authors.

## Ethics Statement

The studies involving human participants were reviewed and approved by Institutional Review Board of Fudan University Shanghai Cancer Center (IRB#090977-1). Written informed consent for participation was not required for this study in accordance with the national legislation and the institutional requirements.

## Author Contributions

Study concepts: FF, ZW, YaZ, and HC. Study design: FF, ZW, YaZ, and HC. Literature research: FF, ZW, ZG, YuZ, YL, YaZ, and HC. Data acquisition: FF, ZW, ZG, YuZ, and YL. Data analysis/interpretation: FF, ZW, ZG, YuZ, and YL. Statistical analysis: FF, ZW, ZG, YuZ, and YL. Manuscript preparation: FF, ZW, YaZ, and HC. Manuscript editing: FF, ZW, YaZ, and HC. Manuscript final version approval: FF, ZW, ZG, YuZ, YL, YaZ, and HC. All authors contributed to the article and approved the submitted version.

## Funding

This work was supported by the National Natural Science Foundation of China (81930073 and 81772466), Shanghai Science and Technology Innovation Action Project (20JC1417200), Shanghai Municipal Science and Technology Major Project (2017SHZDZX01, VBH1323001/026), Shanghai Municipal Key Clinical Specialty Project (SHSLCZDZK02104), and Pilot Project of Fudan University (IDF159045).

## Conflict of Interest

The authors declare that the research was conducted in the absence of any commercial or financial relationships that could be construed as a potential conflict of interest.

## Supplementary Material

Supplementary Figure 1 Overall survival of patients with intraoperatively-confirmed M1a lung adenocarcinoma stratified by cT **(A)** and cN **(B)**.

## Abbreviations

BMI, body mass index; NSCLC, non-small cell lung cancer; OS, overall survival.
