# Supplementary figures and images for: Primary Tumor Resection Improves Survival for EGFR-TKI-Treated Patients With Occult M1a Lung Adenocarcinoma
Source: Front Oncol. 2021 Apr 19;11:622723. doi: 10.3389/fonc.2021.622723 (PMC8092396; doi:10.3389/fonc.2021.622723)

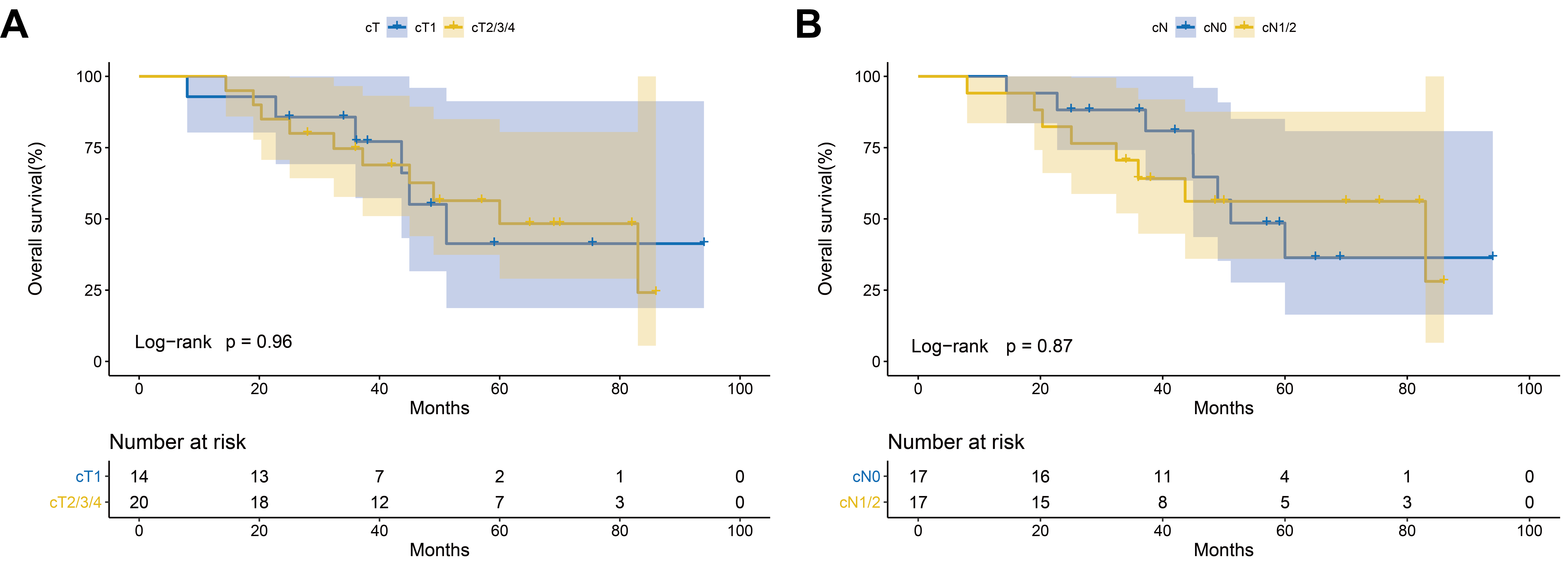

Supplement: Supplementary Figure 1 — Overall survival of patients with intraoperatively-confirmed M1a lung adenocarcinoma stratified by cT (A) and cN (B). [file Image_1.tif]
